# Clinical Implications of Hepatitis B Virus RNA and Covalently Closed Circular DNA in Monitoring Patients with Chronic Hepatitis B Today with a Gaze into the Future: The Field Is Unprepared for a Sterilizing Cure

**DOI:** 10.3390/genes9100483

**Published:** 2018-10-05

**Authors:** Anastasiya Kostyusheva, Dmitry Kostyushev, Sergey Brezgin, Elena Volchkova, Vladimir Chulanov

**Affiliations:** 1 Central Research Institute of Epidemiology, Moscow 111123, Russia; ak@rcvh.ru (A.K.); dk@rcvh.ru (D.K.); sb@rcvh.ru (S.B.); 2 National Research Centre, Institute of Immunology, Federal Medical Biological Agency, Moscow 115478, Russia; 3 I.M. Sechenov First Moscow State Medical University, Ministry of Health of the Russia, Moscow 119146, Russia; az@rcvh.ru

**Keywords:** hepatitis B virus, chronic hepatitis B, pre-genomic RNA, splice variants, covalently closed circular DNA, monitoring, treatment end-points, nucleot(s)ide analogues, interferon, prediction, liver disease

## Abstract

Chronic hepatitis B virus (HBV) infection has long remained a critical global health issue. Covalently closed circular DNA (cccDNA) is a persistent form of the HBV genome that maintains HBV chronicity. Decades of extensive research resulted in the two therapeutic options currently available: nucleot(s)ide analogs and interferon (IFN) therapy. A plethora of reliable markers to monitor HBV patients has been established, including the recently discovered encapsidated pregenomic RNA in serum, which can be used to determine treatment end-points and to predict the susceptibility of patients to IFN. Additionally, HBV RNA splice variants and cccDNA and its epigenetic modifications are associated with the clinical course and risks of hepatocellular carcinoma (HCC) and liver fibrosis. However, new antivirals, including CRISPR/Cas9, APOBEC-mediated degradation of cccDNA, and T-cell therapies aim at completely eliminating HBV, and it is clear that the diagnostic arsenal for defining the long-awaited sterilizing cure is missing. In this review, we discuss the currently available tools for detecting and measuring HBV RNAs and cccDNA, as well as the state-of-the-art in clinical implications of these markers, and debate needs and goals within the context of the sterilizing cure that is soon to come.

## 1. Introduction

Hepatitis B virus (HBV) is a DNA virus that infects human liver and causes acute or chronic hepatitis B (CHB) of variable severity [1,2]. Chronic hepatitis B is characterized by chronic inflammation and the progression of liver disease to fibrosis, cirrhosis, and hepatocellular carcinoma (HCC) [3,4,5]. Epidemiology of CHB is very diverse; its prevalence is estimated to fluctuate around 3–9%, and it is especially common in Asia and the Pacific region [6]. According to recent estimates, over 250 million people are chronically infected and over one million people die annually due to CHB [7]. In particular, CHB-associated HCC accounts for 50% of all liver cancers in the world [3,8,9].

Hepatitis B virus chronicity is mediated by its stealthy nature [10,11], which successfully establishes infection in a non-cytopathic manner, does not induce host immune response in hepatocytes [12], and appears to be generally resistant to intracellular antiviral factors [13]. During infection, HBV establishes a complex replicative cycle that preserves viral persistence in human hepatocytes: covalently closed circular DNA (cccDNA) ensures the maintenance of the virus in hepatocytes, while the replenishment of cccDNA occurs via orchestrated re-import and conversion of the relaxed circular form of the DNA genome (rcDNA) into the nucleus and de novo infection of hepatocytes by HBV virions [14]. De novo infection occurs through packaged, encapsidated HBV rcDNA, and it is potentially also by secreted HBV RNA [15,16]. Moreover, frequent and stochastic integrations of HBV DNA into the host genome using double-stranded linear DNA as a template guarantee stable production of some viral proteins [17,18,19]. Excess HBV surface antigen (HBsAg protein) secreted from infected hepatocytes impairs adaptive immunity and it prevents the immune-mediated resolution of infection [10,20,21].

Modern therapeutics using nucleot(s)ide analogs (NAs) can effectively block reverse transcription of HBV and suppress viral replication [22,23,24]. Moreover, interferon-α (IFN) treatment can achieve sustained antiviral response in a small proportion of patients [24,25,26,27]. These two options represent all the available therapeutics to date, but neither can they completely clear HBV infection in patients. However, these treatments represent valuable and very potent approaches for suppressing viral replication and help significantly to slow down liver disease progression and reduce the risks of adverse CHB outcomes, like cirrhosis and HCC [5,28].

Monitoring cccDNA, including cccDNA epigenetics and mutations, and novel surrogate markers of cccDNA activity, such as secreted HBV RNA, are important for (i) helping to define groups of patients susceptible to IFN therapy [29,30,31]; (ii) determining end-points of NA treatment [16,32]; (iii) predicting the risk of HCC and cirrhosis [33]; and, (iv) identifying potential drug resistance [34]. It should be underscored that in patients receiving these therapeutics, a decline of HBV biomarkers below certain threshold levels testifies to a sustained antiviral response, in which HBV replication is suppressed dramatically and immune cells can effectively control the disease [35,36,37]. While challenges in detecting cccDNA prevent it from being used for monitoring patients with CHB, HBV RNA has been recently proposed as a valuable alternative. It is readily detectable in serum, reflects transcriptional activity and cccDNA levels, and it can be utilized in routine clinical practice [16,38].

One point remains crystal clear: CHB cannot be cured with modern therapeutics. Hepatitis B virus cccDNA persists in hepatocytes and can reactivate HBV infection after cessation of treatment [39,40,41]. Rapid progress in biomedical science promises to provide targeted therapeutics that are aimed at degrading cccDNA and completely clearing the virus in the coming decade. At present, site-specific nucleases CRISPR/Cas9 have been reported to effectively clear HBV cccDNA from the cells [42,43,44,45,46,47,48,49,50]. Apolipoprotein B mRNA editing enzyme, catalytic polypeptide-like (APOBEC) deaminases can be activated by IFNs [51], tumor necrosis factor-α (TNF-α) [52], or CRISPR/Cas9 [53] to induce deamination and degradation of HBV cccDNA while sparing the human genome. Novel approaches in T-cell engineering demonstrated high antiviral activity and complete elimination of the virus in animal models [20,54,55]. It is thus essential to have practical and sensitive methods for defining the point of sterilizing cure, that is, providing evidence that HBV infection is resolved and the virus is completely eliminated from the body. Until now, cccDNA could be detected using ambiguous PCR-based techniques from small liver biopsy specimens, and this detection remains challenging due to technical limitations [56]. Surrogate markers of cccDNA activity and HBV replication are useful in therapy [32,57], but not for determining complete viral clearance with a sterilizing cure at the horizon.

In this review, we summarize recent advances in HBV virology and CHB pathogenesis, discuss pros and cons of cccDNA and HBV RNA monitoring in patients with CHB, and outline the need for a decisive leap to create diagnostic techniques that are capable of identifying complete viral clearance.

## 2. Fundamental Pathobiology of Hepatitis B Virus 

**Figure 1:**
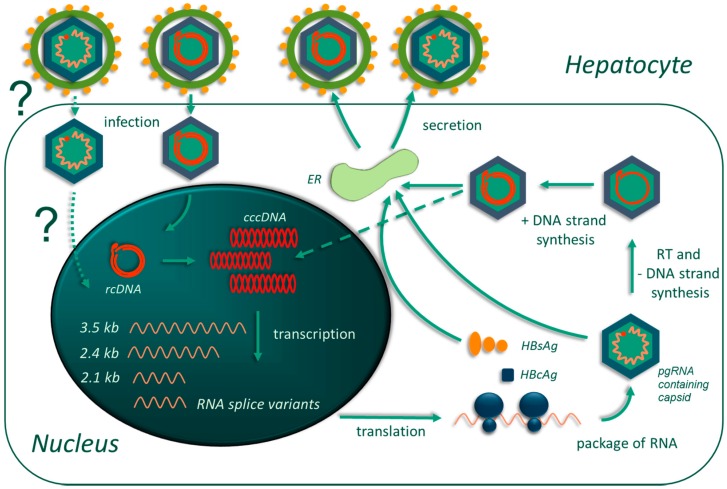
Hepatitis B virus (HBV) life cycle. Hepatocytes are infected by HBV virions containing the relaxed circular form of the DNA genome (rcDNA) or possibly pre-genomic RNA (pgRNA). Viral particles are uncoated and the HBV genome is converted into covalently closed circular DNA (cccDNA), which serves as a template for transcription of all viral RNAs, including spliced RNAs. The major transcript of HBV RNA is pgRNA (3.5 kb), which is reverse-transcribed within nucleocapsids to rcDNA. Hepatitis B virus cccDNA pool is replenished by rcDNA re-imported into the nucleus or by de novo infection. Infected hepatocytes release virions containing rcDNA and RNA, empty virions, and naked capsids (the last two are not shown in this picture). Abbreviations: ER: endoplasmid reticulum; RT: reverse transcription; HBsAg: hepatitis B virus surface antigen; HBcAg: hepatitis B virus core antigen.

Understanding the unique HBV biology is important for adequately conceiving the role of clinical biomarkers in managing CHB patient care and correlating the relevance of these biomarkers to the viral processes occurring in the liver. Therefore, we provide a brief summary of the most important steps in the HBV life cycle relevant to viral persistence and clinical course of the disease (Figure 1). As previously mentioned, HBV is a DNA virus of the family *Hepadnaviridae* that infects mammals, including humans [2,58]. The major form of the HBV genome is rcDNA that has asymmetric DNA strands, which is a unique feature of *Hepadnaviridae*. The length of the negative DNA strand is 3.2 kb, whereas the positive strand varies in size (1.1–2.6 kb). The incomplete positive-strand DNA of HBV provides an essential opportunity for distinguishing cccDNA from other HBV intermediates [59] and for quantitating it using PCR [60] and in situ hybridization techniques [61,62]. Hepatitis B virus produces several types of virions and sub-viral particles, including the so-called Dane particles, which have a lipid membrane containing the HBV surface proteins HBsAg that envelop the nucleocapsid consisting of the core protein HBcAg, viral rcDNA, and the viral polymerase and associated proteins. In addition, it was recently established that HBV produces virions containing pre-genomic RNA (pgRNA) or RNA intermediates, rather than DNA [1,63]. Encapsidated and packaged pgRNA turned out to be a good predictive factor of patients’ responsiveness to antiviral therapy and a promising biomarker for defining therapeutic end-points.

Upon entering the systemic blood flow, HBV virions reach the liver, where interactions of HBV with Na^+^-taurocholate co-transporting peptide (NTCP) receptor via the pre-S1 domain of HBV occurs [64,65,66]. The major function of NTCP is the transport of bile acid anions during enterohepatic circulation [67,68]. Following the infection of hepatocytes, rcDNA in the nucleocapsid is transported into the nucleus to be converted into cccDNA by a multi-step, mechanistically obscure process [69,70,71,72]. A number of factors implicated in the conversion of rcDNA into cccDNA have been recently discovered, but intricate mechanisms of cccDNA generation go far beyond this review. For a detailed investigation of this topic, we suggest one of these excellent reviews [73,74,75]. Hepatitis B virus cccDNA is the template for transcribing all viral RNAs: messenger RNAs (mRNAs) that are translated to viral proteins (large, medium, and small HBsAg; X protein of the hepatitis B virus (HBxAg), involved in regulating cccDNA activity and viral replication; and, hepatits B virus e-antigen (HBeAg)) and pgRNA, which encodes HBcAg, the viral polymerase, and a precursor of rcDNA. All types of HBV RNAs (subgenomic, pre-genomic, and pre-core RNAs) are transcribed by RNA polymerase II and they are capped at 5′-termini and polyadenylated at 3′-termini [76] and are therefore readily translated by the cell. Hepatitis B virus pgRNA, together with the viral polymerase and cellular proteins, is encapsidated into de novo-synthesized viral nucleocapsids [77,78]. Next, pgRNA is reverse-transcribed to rcDNA within these nucleocapsids and is simultaneously degraded by RNase H activity of the viral polymerase [79]. Hepatitis B virus rcDNA can be generated only from pgRNA [80].

## 3. Chronic Hepatitis B: Chronicity, Clinical Course, and Clinical Markers

Hepatitis B virus persistence in human hepatocytes is caused by several complementary factors: (a) cccDNA safely persists in cell nuclei [81]; (b) the intracellular host immune response is significantly impaired by the virus [20]; and, (c) HBV effectively evades immune control by immune-competent cells [20]. 

Although it was previously suggested that HBV cccDNA may persist in hepatocytes for years [82] or for the life-span of non-dividing cells [83], a more recent report suggests that the half-life of cccDNA could be as short as 40 days in HepG2 cells [14]. Stability of the HBV cccDNA pool is ensured by the replenishment of cccDNA both by newly produced rcDNA in hepatocytes and secondary de novo HBV infection. Inhibition of HBV replication by NAs significantly suppresses HBV replication and reduces the cccDNA pool size, but the common outcome after cessation of therapy is viral reactivation [37,40,41]. Another reason for HBV persistence is its capability to evade host immune responses [13,84,85]. Hepatitis B virus does not induce an IFN response in acutely infected and CHB patients, although several reports indicate moderate activation of IFN-λ response in several systems [86]. Hepatitis B virus has been shown to abrogate IFN response in a plethora of studies [87,88,89], although several recent reports argue this notion [13,84,90]. Moreover, HBV infection establishes an immunosuppressive state, reducing absolute amounts and suppressing function of HBV-specific T-lymphocytes [91], NK-cells [92], dendritic cells [93], and others. It was reported that HBV targets the adaptive immune response to increase its survival and replication in hepatocytes [20]. Overall, pathogenesis and outcomes of HBV infection are defined by the strength of the antiviral immune response [94]. 

Based on the levels of alanine aminotransferase (ALT), serum HBV DNA, and HBeAg status, chronic HBV infection is divided into four conventional phases: (i) the immune-tolerant phase, (ii) the HBeAg-positive immune-active phase, (iii) the inactive carrier phase, and (iv) HBeAg-negative immune-active phase [95]. These phases do not necessarily follow each other but can switch in a non-linear fashion. The immune-tolerant phase is actually a low-inflammatory phase that is characterized by the presence of HBeAg, high serum HBV DNA and HBsAg levels, and normal ALT levels. After this phase, CHB patients enter the HBeAg-positive immune-active phase, which is characterized by profound liver inflammation and elevated ALT levels. Upon the seroconversion of HBeAg-positive patients to the HBe-antibody positive state, some patients enter the inactive carrier phase wherein HBV DNA and HBsAg remain low or even undetectable and liver inflammation subsides (normal ALT). Moreover, a recent study demonstrated that proportions of HBV large and middle surface proteins were significantly lower in inactive carriers than in CHB patients, and thus the quantification of large and middle surface proteins may serve as a valuable tool to distinguish inactive carrier from other CHB patients [96]. In some cases, ALT and HBV DNA levels elevate again after years of inactive carrier state. In HBeAg-negative patients, HBsAg serves as a particularly important biomarker for determining the phase of infection and predicting the risk of serious CHB outcomes in patients with low viremia. A separate group of patients includes those who spontaneously clear HBsAg, and, in some cases, anti-HBs antibodies. These patients are considered to have occult HBV infection, as characterized by the disappearance of HBsAg and of most HBV DNA in serum. However, it must be underscored that in every phase of CHB, cccDNA persists in hepatocytes and cannot be eliminated by currently available therapeutic options [73]. In theory, a single remaining copy of cccDNA may suffice to restart HBV infection in the liver [97]. 

## 4. Therapeutic Options for Chronic Hepatitis B: Raiders of the Deceptive Cure

Prior to discussing different types of CHB therapy, it is necessary to clearly understand what is meant by the cure of CHB. Currently, the understanding of CHB cure and therapeutic end-points is the subject of active debates. In an ideal situation, a cure is the complete loss of viral intermediates and the clearance of every copy of HBV cccDNA from the liver. In this case, infection relapse after the cessation of antiviral therapy does not occur. However, this type of a complete, or sterilizing, cure cannot be achieved by currently available drugs [95]. Another therapeutic goal is the so-called functional cure [98,99,100], which is a rare state characterized by sustained anti-viral response, elimination of HBsAg, and a significant decline in serum HBV DNA below the limit of detection. The hallmark of this type of cure is a lasting or complete immunological control of HBV infection. The major advantages of this goal are that in rare cases it can be achieved by modern antivirals and can be easily monitored. However, stopping antiviral therapy induces the reactivation of HBV infection and full-blown CHB [39,41,101,102]. 

Another alternative therapeutic goal is a para-functional cure [57,103]. This term was coined in 2016 after the discovery of pgRNA in HBV virions secreted from infected hepatocytes in the serum [38]. Those findings strongly suggested that in some cases, long-term NA treatment converts cccDNA into a transcriptionally inactive form that is characterized by the abrogation of pgRNA secretion in the serum. No relapse was seen in 50% of the patients who achieved this para-functional cure. Apparently, the relatively high incidence of HBV reactivation in this group of patients is attributed to the low sensitivity of available methods for detecting pgRNA in human serum [103]. Nevertheless, it is evident that transcriptional repression of cccDNA cannot be considered a proper cure, as the virus persists in hepatocytes, and risks of HCC and fibrosis remain elevated. 

At the moment, a functional cure remains the preferable therapeutic goal, but this type of cure is deceptive because it does not imply that the patient is free of HBV, as cccDNA persists in the liver, nor does it eliminate the risk of HCC development, as cccDNA in hepatocytes produces viral HBx and preS-containing HBs proteins with oncogenic potential [104]. HBx protein is one of the major causes of HCC, as it functions as a transactivator of proto-oncogenes in the cells, produces reactive oxygen species, and induces DNA damage and genomic instability [105,106,107,108]. HBsAg is produced both from episomal cccDNA and viral DNA integrations, and it serves as an additional oncogenic factor, promoting cell proliferation and transformation [17,109]. Thus, elimination of HBsAg and transcriptional silencing of cccDNA and seroconversion cannot be regarded as markers of a cure, but rather as states with reduced viral activity and relative immune control.

Currently, two main therapeutic options are available to treat CHB: NAs (adefovir [110], entecavir [111], lamivudine [112], telbivudine [113], and tenofovir [23,114]), which inhibit viral reverse transcriptase, and standard or pegylated IFN-α (PEG-IFN) therapy [115]. Adefovir and lamivudine are currently not used in clinical practice due to low antiviral activity and the rapid development of drug resistance [116,117]. The primary goal of current treatment is sustained anti-viral response.

Treatment with NAs for over one year reduces serum HBV DNA by more than four logs, while intrahepatic cccDNA decline is less prominent (1 log). Among NAs, entecavir and tenofovir suppress HBV replication most potently. Treatment with entecavir over a five-year period maintains or suppresses serum HBV DNA (<300 copies/mL in 94% of CHB patients), normalizes ALT levels in 80% of patients, and results in HBeAg loss and seroconversion [118]. Notably, entecavir resistance emerged in 1 out of 183 patients [119]. Additionally, utility of entecavir is very limited in lamivudine-resistant patients, as rapid selection of HBV mutants resistant against entecavir occurs [120]. Results from clinical trials of tenofovir reported even higher antiviral activity: in an eight-year phase III study, sustained anti-viral response was achieved in 98% of HBeAg-positive and 99% of HBeAg-negative patients. Tenofovir sisoproxil fumarate (TDF) treatment is well tolerated over long time periods, and no resistance emerged in patients that were undergoing this treatment [121]. However, risks of adverse effects, including risks of nephrotoxicity, upon TDF treatment were elevated in certain groups of patients (e.g., HBV/HIV co-infection) [122]. A second-generation pro-drug, tenofovir alafenamide (TAF or GS-7340) is more stable, can be prescribed at lower doses than TDF [123], and demonstrates high antiviral activity and low toxicity in clinical trials [124]. 

Therapy with NAs results in seven times higher five-year survival rates in patients with CHB [125]. Moreover, treatment with NAs can stop or even reverse the progression of CHB to fibrosis and cirrhosis [125,126]. Also, NAs can reduce the severity of portal hypertension [127,128] and the risks of HCC development [129].

On the other hand, IFN therapy also leads to a sustained antiviral response, but only a minority of CHB patients (~30%) respond to IFN treatment [130]. Treating CHB patients with PEG-IFN for 48–52 weeks results in HBeAg seroconversion in 24–27% of the patients and to HBsAg loss in 3–7% of the patients, while over the same period, NAs lead to HBeAg loss in 12–22% of patients and HBsAg loss in 0–3% of patients [131]. In addition, treatment with IFN frequently causes severe side effects and it is more poorly tolerated than NAs [132,133].

Although it was previously shown that cccDNA is very stable in non-dividing human hepatocytes, where it appears to survive for the life span of the cell [83], the half-life of cccDNA in dividing cells was recently defined in several studies. These results showed promise, as the half-life in dividing cells turned out to be relatively short [14,134]. Since NAs can effectively inhibit HBV reverse transcription and reduce viremia below detection limits and even decrease intrahepatic cccDNA levels by inhibiting cccDNA replenishment, it was suggested that long-term treatment could lead to a cure of CHB. However, cccDNA persists in hepatocytes even after years of drug administration, and cessation of antiviral treatment induces relapse of viral activity. On the other hand, restoration of immune anti-HBV responses after stopping long-term NA treatment could contribute to the clearance of HBV infection. Indeed, after stopping NA treatment, patients acquired better immunological control over the disease, and they were reported to have reduced levels of intrahepatic cccDNA and viral replication [135].

Several recent breakthroughs in biomedical research paved the way for the development of direct-acting antivirals capable of cleaving and degrading cccDNA (Table 1). First, adapting the bacterial immune system CRISPR/Cas9 enables the precise and efficient exhaustion of HBV cccDNA. A plethora of in vitro and in vivo studies proved high antiviral activity of CRISPR/Cas9, showing substantial decline in cccDNA and clearance of HBV intermediates [42,43,44,45,47,136,137]. Inducing expression of deaminases APOBEC3A and APOBEC3B in hepatocytes by IFN [51] or dCas9 activation tools [53] enables the non-cytopathic degradation of cccDNA. APOBECs are recruited to cccDNA by interaction with HBcAg and deaminated G/C nucleotides throughout cccDNA. Widespread deamination is followed by degradation of cccDNA and significant suppression of HBV replication. T cells with chimeric antigen receptors and different approaches for stimulating adaptive immunity have been reported to kill infected cells and resolve HBV infection in animal models [54,55]. Although all of these approaches still need to be tested for safety and tolerance by CHB patients, the experimental background behind these techniques is astonishing. In summary, a battery of new antivirals with direct cccDNA-targeting properties will soon enter different phases of clinical studies. These antivirals provide an unprecedented opportunity to destroy HBV cccDNA and completely erase the virus, but new achievements open up new challenges, and the main challenge yet unresolved is the non-invasive but reliable detection of cccDNA and other HBV intermediates in the human body. 

## 5. Covalently Closed Circular DNA Levels: A Marker for Predicting Antiviral Response 

Entry of HBV into the cell and transportation of rcDNA into the nucleus establishes the cccDNA pool that ensures persistent infection [73]. cccDNA is the crucial intermediate that cannot be targeted by currently available drugs and thus represents the key obstacle to curing CHB. Nucleot(s)ide analogs inhibit the formation of new virions, but HBV RNA synthesis and protein translation remain unperturbed. Despite the profound decline in cccDNA replenishment by rcDNA reimport into the nucleus during NA treatment, cccDNA levels decline very slowly in CHB patients [97]. The actual copy number of cccDNA in infected hepatocytes and how cell division or adaptive immune responses reduce copy numbers in CHB patients remains largely unknown.

However, it is certain that, irrespective of the phase of HBV infection, the type of anti-HBV therapy or its duration, cccDNA is not eliminated from liver cells and can be detected even after HBsAg and HBV DNA disappear from serum [138,139]. As single copies of cccDNA are thought to start a full-blown infection, only complete elimination of cccDNA can be considered as a complete cure without the risk of viral reactivation [140]. However, despite years of investigation and a number of highly effective medications, the complete elimination of cccDNA in hepatocytes is not yet possible. 

It is worth noting that intrahepatic cccDNA levels vary widely (0.003–6.8 copies/cell) in patients depending on the phase of the disease, viral replication, HBV genetics, and other factors. Clinical data demonstrate that HBV cccDNA levels correlate with HBeAg status and viral replication. The highest quantities of cccDNA (1–40/cell) are detected in HBeAg-positive patients with elevated total intracellular (95–9.890 copies/cell) and serum HBV DNA (107–109 copies/cell). In comparison, cccDNA levels are ten times lower in HBeAg-negative patients [138,139]. Hepatitis B virus cccDNA levels were reported to be less than 0.1 copies/cell in HBeAg-negative patients with low viremia [140]. NA therapy for over 48 weeks reduced cccDNA levels by more than 85% [141].

Interferon therapy leads to HBeAg seroconversion in only a small proportion (about 30%) of HBeAg-positive CHB patients [115]. Since IFN therapy is notoriously toxic and it causes significant side effects, it is important to define reliable biomarkers to predict IFN responsiveness soon after the start of treatment. Importantly, cccDNA levels were recently reported to be a predictive factor of success of IFN therapy [30,142]. In a recent study [30] with HBeAg-positive patients receiving IFN therapy, HBV DNA and cccDNA significantly declined in IFN responders when compared to non-responders. Most importantly, baseline values of intrahepatic HBV DNA over cccDNA in liver specimens significantly correlated with IFN responsiveness. Moreover, patients with transcriptionally active cccDNA (higher ratios of HBV DNA/cccDNA) turned out to be more susceptible to IFN-mediated decline in cccDNA than patients with transcriptionally-inactive cccDNA (lower ratios of HBV DNA/cccDNA) [30]. Thus, cccDNA quantification could serve as a reliable marker to predict patients’ response to IFN therapy and to assess treatment efficacy [143,144]. 

## 6. Covalently Closed Circular DNA and Adverse Chronic Hepatitis B Outcomes 

As outlined earlier, HBV cccDNA plays an important role in HBV replication and persistence. In addition, cccDNA can be used to monitor disease progression and to predict the risk of adverse CHB outcomes. Chronic liver inflammation induced by HBV infection leads to fibrosis, cirrhosis, and HCC [94], one of the leading causes of death in CHB patients. Although correlation between HBV cccDNA in the liver and liver inflammation has been largely controversial in published literature [91,145], higher levels of intrahepatic HBV cccDNA positively correlated with serum ALT levels and were clearly shown to be associated with more severe liver inflammation in HBeAg-positive patients [33]. A study with treatment-naive patients demonstrated that more than one copy of cccDNA per cell and liver inflammation grade ≥ 1 are independent prognostic factors for the risk of liver inflammation, and the authors infer that higher baseline levels of HBV cccDNA might significantly increase the risk of liver inflammation. Moreover, increased HBV DNA/cccDNA ratio is related to active viral replication and is associated with liver disease progression and poor disease outcomes [33].

As HBV cccDNA exists as a mini-chromosome complexed with histone and non-histone proteins, its transcriptional activity is prone to epigenetic modifications [146]. A growing body of evidence suggests that methylation of HBV cccDNA plays an important role in regulating cccDNA transcription [147,148,149,150,151]. Three canonical CpG islands in the HBV genome can be methylated by DNA methyltransferases (DNMT) of the host cells [148,149]. Methylated HBV DNA has been identified in blood serum and biopsies from CHB patients [150], and mRNA levels of DNA methyltransferases DNMT1, DNMT3A, and DNMT3B were elevated in these specimens [152]. Methylation of CpG islands in HBV cccDNA diminishes viral protein production [153]: hypermethylation of CpG islands decreases HBsAg and HBeAg levels [150], as well as levels of viral mRNAs [154]. Increased expression of DNMTs could be a part of non-specific intracellular anti-HBV response released by infected cells to dampen viral transcription [155]. However, the methylation of the HBV genome is not specific, so the host genome of patients with CHB becomes a target for undesirable DNA methylation [156,157]. Methylation of functionally relevant host genes leads to the dysregulation of cellular processes and may inflict DNA damage. Hepatitis B virus itself and an HBV-related increase in DNMT expression aggravate genome damage and accelerate development of fibrosis and HCC. Indeed, methylation status of HBV CpG islands correlates with the stage of liver fibrosis. According to Zhang et al. (2014), cccDNA CpG island I, which is generally unmethylated, becomes methylated in patients with late-stage liver fibrosis, while median methylation rates in CpG islands II and III are significantly higher in patients with Knodell fibrosis stage 3–4 [148]. The authors also identified that the HBV genotype, HBeAg positivity status, and patient age correlate with cccDNA methylation. To conclude, cccDNA methylation has not been shown to serve as a predictive factor of liver disease progression and it rather represents the severity of liver disease progression. 

## 7. Hepatitis B Virus RNA in Secreted Virions

An immense leap in our understanding of HBV biology came with the discovery of encapsidated and enveloped HBV RNA secreted from infected cells [38]. The majority of circulating HBV virions in human sera contains rcDNA, but recent reports demonstrated that certain serum HBV virions contain HBV RNA [15,16]. The nature and origin of this RNA have not been extensively studied yet, nor is it known whether these packaged HBV RNAs contribute to the infection process and viral spreading. However, secreted HBV RNAs have already been established as a valuable serological marker in CHB patients [24,29,34]. Potential utility of HBV cccDNA and HBV RNA in the clinic is outlined in Table 2. 

Secreted HBV virions have long been known to contain not only rcDNA, but also HBV RNA [158]. During HBV infection, viral pgRNA is encapsidated and it serves as a template for synthesizing rcDNA [38]. Maturation and envelopment of the viral capsid were believed to rely on pgRNA-dependent rcDNA synthesis; thus, the secretion of pgRNA-containing capsids should not occur. However, several recent studies showed that viral capsids can mature independently of HBV DNA synthesis [159,160]. Interestingly, RNA-containing virions are more frequent in patients receiving NAs [16]. Most likely, the explanation of this phenomenon could be the excessive accumulation of pgRNA that is not reverse-transcribed so that rcDNA is not generated, leading to the generation of HBV virions without viral DNA.

Currently, correlations between serum HBV RNA levels and clinical parameters are actively investigated, because they can purportedly reveal cccDNA transcriptional activity [16,161]. In particular, average serum HBV RNA levels were reported to be higher in HBeAg-positive (6.5 log c/mL) than in HBeAg-negative (5.9 log c/mL) patients. Factors that are associated with low serum HBV RNA include high ALT, HBeAg-negative status, HBV genotypes A, B, and C, and the presence of HBV basal core promoter (BCP)/pre-core mutations (Table 3). Serum HBV RNA was correlated with HBV DNA in HBeAg-negative and HBeAg-positive patients, and with HBsAg in HBeAg-positive patients (Table 4) [162].

Serum HBV RNA could become a valuable biomarker for predicting tyrosine-methionine-aspartate-aspartate (YMDD) mutations of drug resistance [34], predicting responses to therapy [163,164], and defining CHB treatment end-points [165]. Hatakeyama et al. (2007) showed that CHB patients with high serum HBV RNA levels receiving lamivudine developed drug resistance in the first year of treatment, while patients with low serum HBV RNA developed drug resistance much later, if at all [34]. In this study, HBV RNA was concluded to be transcribed mainly from the integrated HBV genome, as it did not correlate with HBV DNA. Other studies revealed that the HBV genotype, ALT level, HBV DNA levels prior to therapy, the magnitude of HBV DNA decline upon antiviral therapy, presence of HBeAg, and HBV DNA mutations may serve as predictive factors of the emergence of YMDD mutants. This study provides evidence that routine monitoring of HBV RNA levels might serve as a better alternative to predict emergence of YMDD mutants.

Uptake of NAs can significantly suppress HBV replication and reduce serum HBV DNA, but complete cure of CHB cannot be achieved due to persistence of cccDNA. NAs do not directly target cccDNA, but the cccDNA pool can be diminished indirectly by the reduced replenishment of cccDNA due to rcDNA import [1]. Nucleot(s)ide analog treatment can result in disappearance of serum HBV DNA, but pgRNA-containing HBV virions continue to be secreted [161]. Detection of pgRNA-containing virions may reflect the level of transcriptionally-active cccDNA. It was suggested that undetectable serum pgRNA can serve as an important end-point for stopping NA treatment [16]. To stop antiviral NA therapy, it is necessary to guarantee that cccDNA is transcriptionally inactive and that withdrawing the antivirals will not lead to HBV relapse. Thus, serum pgRNA is a valuable surrogate marker of cccDNA, which can be utilized in clinical practice to predict patients’ responses to therapeutics and to define treatment end-points.

One realistic but hard to achieve stopping rules in CHB patients is HBsAg loss. However, NA therapy can be stopped even if HBsAg is detected in serum [166]. In CHB patients, HBV DNA integrates into the host genome, and in HBeAg-negative patients, HBV DNA integrations may preferentially serve as templates for producing HBsAg. Therefore, HBsAg in this case is not related to cccDNA activity, but rather it reflects HBsAg production by infected cells [167]. An alternative biomarker for stopping antiviral therapy in HBeAg-negative patients is low or undetectable level of serum HBV DNA, but this is not reliable, as HBV frequently relapses soon after NA discontinuation [168]. Hepatitis B virus RNA in serum was proposed as an additional biomarker of cccDNA transcriptional activity to define sustained anti-viral response in HBeAg-negative patients [165]. Serum HBV RNA was also suggested to predict responses to IFN therapy in CHB patients [29]. Van Bommel et al. (2018) demonstrated that measuring serum HBV RNA in patients receiving PEG-IFN-α-2a helps to predict HBeAg seroconversion [29]. It is particularly important to identify the patients who are not susceptible to IFN therapy because of the severe adverse effects of IFN and low rates of IFN responsiveness. Several biomarkers were previously associated with IFN responsiveness, including HBV genotype A, high ALT levels, low HBV DNA, HBeAg, and HBsAg levels in serum [169,170]. However, these parameters cover a very small proportion of CHB patients. In recent studies, HBV RNA levels were profoundly lower in patients who further underwent HBeAg seroconversion upon IFN therapy [171]. Hepatitis B virus RNA serum levels > 5.5 log_10_ copies/mL at 12 and 24 weeks of IFN therapy allowed the identification of 30% of non-responders. Serum HBV RNA was found to predict IFN responsiveness not only in HBeAg-positive, but also in HBeAg-negative patients. High levels of serum HBV RNA at week 12 were a reliable marker of not responding to IFN therapy [171].

## 8. Hepatitis B Virus RNA Splice Variants in Disease Progression and Outcomes

Constitutive splicing is an important step for regulating gene transcription in cells [172]. Alternative splicing contributes to generation of divergent eukaryotic proteins and creates another level of regulatory control over gene expression [173]. Several decades ago, HBV transcripts were shown to undergo alternative splicing in vitro and in CHB patients [31,174,175]. The major HBV transcript (pgRNA) can be alternatively spliced to generate several different splice variants, including SP1RNA (RNA splice variant with one-third of the viral genome deleted), which accounts for over 30% of total HBV pgRNA [176]. Similar to pgRNA, SP1RNA can be packaged to generate defective HBV circulating particles. The relevance of HBV RNA splicing in CHB pathogenesis and the clinical course of disease has remained mostly elusive, but recent reports demonstrated that defective HBV particles gradually increase with severity of liver inflammation [177,178,179]. Defective HBV particles may constitute up to 69% of total serum virions [180]. Several clinical studies unveiled a link between defective HBV particles, viral load, and liver disease progression [177,181]. In experimental models of humanized mice, chemical liver damage resulted in a significant increase of HBV splice variants. Average levels of HBV splice variants are higher in patients with severe liver necrosis and fibrosis [180]. Purportedly, HBV spliced variants enhance promotion of HBV replication by the X gene [182]. Several HBV RNA transcripts were reported to promote cell cycle progression and they are likely to have pro-oncogenic activity [183].

Elevated proportions of HBV splice variants in serum of CHB patients were shown to negatively correlate with response to IFN-α treatment [31]. These splice variants of HBV RNA can be translated into proteins harboring domains that could counter IFN signaling in host cells [31]. To decipher the underlying mechanisms, three major splice variants of HBV RNA were transfected into the cells and were shown to strongly suppress IFN-α signaling. Thus, increased proportions of HBV splice variants are associated with dysregulated IFN signal transduction and the resistance of CHB patients to IFN therapy. Moreover, different HBV genotypes have different levels of splice variants [31,184]. Perhaps distinct levels of spliced RNAs in different HBV genotypes reflect distinct preferences of each HBV genome to generate splicing patterns. Hepatitis B virus genotype D expresses the highest levels of spliced RNA, followed by genotypes C, B, and A; these levels might reflect clinical responses of CHB patients to IFN-α therapy in the reverse order, with HBV D patients being least responsive.

Detection of HBV DNA splice variants in the serum reflects the levels of intracellular HBV RNA splice variants. Therefore, the identification of HBV DNA instead of intrahepatic HBV RNA is an amenable tool for predicting IFN responsiveness. At the same time, analysis of HBV RNA splice variants in secreted HBV virions has not been investigated, but it could contribute to diagnostic advances for better selecting patients for IFN therapy. Overall, monitoring abundance of HBV RNA splice variants in serum may also help to reveal groups of patients susceptible to IFN-α therapy. 

## 9. Methods and Challenges of Detecting cccDNA and pgRNA 

Hepatitis B virus cccDNA is the crucial form of HBV genome that is responsible for the persistence of the virus in human hepatocytes. Eliminating cccDNA could be the primary goal of antiviral therapy and one of the most important biomarkers for predicting and monitoring patients’ response to antiviral therapy, as well as assessing the risk of liver disease progression. The major obstacle to monitoring cccDNA in these settings is the lack of sensitive and unambiguous methods to detect cccDNA, which remains one of the biggest challenges in the field of hepatology [56]. All modern tools are based on detecting cccDNA in liver biopsy specimens [60], representing a small piece of the liver in which cccDNA could be extremely or even undetectably low in CHB patients, especially those that are undergoing antiviral therapy [97]. 

Among the available arsenal of instruments (summarized in Table 5), one of the most widely accepted and reliable methods is Southern blotting, which can unequivocally determine and perform a semi-quantitative analysis of cccDNA. However, the extremely low sensitivity and time-consuming process of this technique limit its use [185]. The much more sensitive PCR-based techniques (real-time PCR [186], droplet-digital PCR [60], competitive PCR, and Invader Assay) [187], coupled with novel approaches for cccDNA isolation and enrichment (modified Hirt procedure [188], T5/PSAD DNase digestion of linear and chromosome DNA [189,190], and magnetic separation of cccDNA [191]) are among the available tools for quantifying cccDNA from liver biopsies with a certain degree of confidence [56,69]. Advantages and downsides of these techniques have been described elsewhere [56,192], but the common concern is that they might underrepresent cccDNA levels or amplify other forms of the HBV genome, such as mature rcDNA particles or HBV DNA integrants, along with cccDNA. Recent technological developments provided an exciting opportunity to visualize cccDNA templates in specially prepared tissue samples and cell cultures, including fluorescent in situ hybridization (FISH) detection of cccDNA [61,134]. The latter was shown to be highly sensitive (over 8000-fold amplification) and specific to probe cccDNA, distinguishing it from other genomic forms. However, visualizing cccDNA remains impractical due to difficult sample preparation and complex equipment needed to monitor cccDNA in a clinical setting. While PCR-based techniques to quantify cccDNA remain the only option available for clinical use, rapid progress in development of medications aimed at complete elimination of the virus and curing CHB requires a new paradigm for non-invasively detecting cccDNA in the whole liver of patients. 

Throughout this review, it has been emphasized that a single copy of cccDNA remaining in a patient may, in theory, start a full-blown infection process and compromise the efforts to cure CHB [73]. The only possible end-point for a complete cure, then, must be a complete elimination of the virus, including cccDNA. This requires creation of a novel approach to visualize cccDNA in the human body. The major challenge of such an approach is that the detection of cccDNA existing in a complex with various proteins is not feasible with modern techniques. First and foremost, cccDNA strands must be unwound to provide a signal that is readily detectable by biomedical instruments with high sensitivity. The technique may not even need to distinguish cccDNA from other forms of the HBV genome, as a sterilizing cure implies complete clearance of all forms of the virus from the body. The most promising candidates for developing such a technique are CRISPR/Cas9 visualization tools. Cas9 proteins can be recruited to designated locations in the viral genome, and attaching a reporter to the Cas9 protein provides a specific, strong signal corresponding to the desired target [193]. To date, this technique has been utilized to visualize repetitive sequences in the human genome in vitro or any genomic sequence using a battery of guide RNAs for Cas9 proteins [194,195,196,197,198]. Obviously, new technological breakthroughs are essential to advance this technique and adapt it for CHB patients, and many obstacles need to be overcome, e.g. potential immunogenicity of the Cas9 protein, mechanism of signal detection, and distribution and delivery of Cas9/gRNAs ribonucleoprotein complexes. 

Alternatively, several surrogate markers (HBeAg, serum HBV DNA, HBsAg, and secreted HBV RNA) have been applied to assess the efficacy of antiviral therapy and define the treatment end-points [57,199]. These methods demonstrated high sensitivity, specificity, and applicability in certain clinical settings. For example, the quantitation of secreted HBV RNA has been used to predict susceptibility of CHB patients to IFN-𝛼 therapy [24,31,184], to determine the functional and para-functional cure, and pinpoint when discontinuing NA treatment would not lead to relapse. Secreted HBV RNA can be amplified by RACE-PCR techniques, or by standard PCR after degrading viral DNA using DNase I [16]. RACE-PCR allows for the specific amplification of HBV RNAs without additional steps in the isolation procedure and it is quite sensitive; protocols relying on DNase I treatment require the purification of the isolate to remove DNase, as the enzyme may compromise the subsequent PCR reaction. Enrichment of RNA as compared to isolated DNA by extraction on silica resins leaves out significant amounts of HBV DNA and it cannot be used for accurate HBV RNA detection [200]. A recent kit that was developed by Abbott (Abbot Park, Illinois, United States) utilizes a high-throughput assay that can detect HBV RNA with a 44 U/mL limit of detection [201]. In comparison, the reported limit of detection for RACE-PCR is ~54 U/mL. 

The above-described surrogate biomarkers are valid and can be readily detected, but they may reflect only transcriptional silencing of cccDNA or immunological control of HBV infection. 

## 10. Conclusions

In conclusion, intracellular cccDNA and its novel surrogate marker, secreted HBV RNA, are important biomarkers, as they point to the replicational activity of the virus and they can be used to address various clinical needs. However, technical limitations and ethical issues do not allow for using intrahepatic cccDNA for actively monitoring patients with CHB. Serum HBV RNA can be readily detected by very accurate and sensitive methods that are widely used in diagnostic laboratories. Good correlation of HBV RNA with other HBV biomarkers, and its strong association with different parameters of CHB make it an amenable tool that can improve CHB management. Given rapid progress in HBV research, we envision the development of a CHB cure within the current decade. An absolute cure implies the complete clearance of HBV from the human body. Therefore, it is important to apprehend that current diagnostic techniques cannot define the state of an absolute cure. Hepatitis B virus cccDNA detection from liver biopsies and surrogate markers cannot meet the needs of the very near future. 

## Figures and Tables

**Table 1:** Novel therapeutics for chronic hepatitis B (CHB).

Therapeutic Agents	Representative Drugs	Phase of Clinical Trials
Inhibition of HBV entry	Myrcludex-B	Phase II clinical trials
Degradation of cccDNA	CRISPR/Cas9, APOBEC-deaminases, LT-βR agonist	Preclinical studies
Capsid assembly inhibitors	GLS4, NVR 3-778, AIC 649, ABI-H0731	Phases I-II clinical trials
miRNA	ARB-1467, ARB-1740	Phase II clinical trials
Therapeutic vaccinations	INO-1800, HB-110, TG1050, HepTcell	Phase I clinical trials
Intracellular immune response agonists	GS 9620, SB9200, AIC649	Phase II clinical trials
cccDNA inhibitors	CCC-0975, CCC-0346	Preclinical studies
HBsAg inhibitors	Rep 2139, Rep 2055	II phase of clinical trials

**Table 2:** Clinical utility of HBV cccDNA and RNAs.

Marker	Applications	Result
**cccDNA**	Defining absolute cure	cccDNA is undetectable.
	Predicting IFN responsiveness in HBeAg-positive patients	cccDNA level is lower in IFN responders than non-responders.
**Serum HBV RNA**	Safely discontinuing NA therapy	HBV RNA is undetectable.
	Predicting YMDD mutations	High serum HBV RNA levels predict lamivudine resistance after the first year of treatment.
	Predicting HBeAg seroconversion in HBeAg-positive patients receiving IFN	HBV RNA levels > 5.5; log_10_ copies/mL predict non-responders to IFN therapy (weeks 12 and 24).
	Predicting IFN responsiveness in HBeAg-negative patients.	High levels of serum HBV RNA are a reliable marker of non-responsiveness to IFN therapy (week 12).
**HBV RNA splice variants**	Predicting IFN responsiveness	Elevated HBV splice variants in serum negatively correlate with responsiveness to IFN treatment; HBV DNA splice variants in the serum reflect the levels of intracellular HBV RNA splice variants.

**Table 3:** Factors affecting HBV RNA levels in serum.

Factors	Effect
Presence of BCP variants	Lower HBV RNA serum levels
HBV genotype	Patients with HBV of genotypes A, B, and C have lower HBV RNA serum levels than of genotype D
ALT levels	Higher in patients with ALT level > 2 × upper limit of normal (ULN) compared to patients with ALT level < 2 × ULN
Patient’s age	No influence
Patient’s sex	No influence

**Table 4:** Comparison of HBV RNA parameters in HBeAg-positive and HBeAg-negative patients.

Parameter	HBeAg-Positive	HBeAg-Negative
Mean serum HBV RNA level	6.5 (1.2) log c/mL	4.1 (1.2) log c/mL
Correlation of serum HBV RNA and HBV DNA	Strong	Strong
Correlation between HBV RNA and HBsAg	Moderate	Weak

**Table 5:** Comparison of modern methods to detect and quantify HBV cccDNA and pgRNA.

	Method	Specificity	Limit of Detection	Advantages	Disadvantages
**cccDNA**	Southern blotting	Unequivocally determines cccDNA	2 × 10^6^ copies	Reliable; reproducible	Complicated; costly; time-consuming; safety concerns
	Conventional qPCR	May under- or overrepresent cccDNA	2 × 10^3^ copies/mL	Simple; rapid; accurate; economical, sensitive	Lower specificity when rcDNA is abundant
	Competitive qPCR	More specific than conventional qPCR; may still overrepresent cccDNA by amplifying rcDNA	2 × 10^4^ copies	More specific and accurate than conventional qPCR; readily distinguishes cccDNA from rcDNA	Lower specificity when rcDNA is abundant
	Droplet-digital PCR	Specific	1 copy; upper detection limit is restricted	Super-sensitive; accurate	Detection is impaired when cccDNA number is greater than 10^6^ copies
	Rolling circle amplification qPCR	Specific	10^2^ copies/mL	Practical; sensitive; specific	Time-consuming; cross-linked proteins impair effective amplification
	Rolling circle amplification-in situ qPCR	Highly specific; cross-linked proteins could hinder effective amplification	2 copies/cell	cccDNA detection at single-cell resolution	Diffusion of amplified DNA to neighboring cells; cross-linked proteins impair effective amplification
	Magnetic capture hybridization qPCR	Specific	90 IU/mL	Specific	Does not capture all cccDNA; complicated; costly
	Invader assay	Specific; minimal interference from double-stranded and integrated HBV DNA	50 copies (10^4^ copies/mL)	Provides a specific and simple method for detecting cccDNA comparable with PCR	Interference from rcDNA and integrated HBV DNA
	FISH detection	Specific; distinguishes cccDNA at single-cell resolution; no diffusion of amplified products	1 copy under optimal conditions	Specific; visible at single-cell resolution; can distinguish and locate various DNA, RNA and proteins; without diffusion of amplified products	Complicated probe design
	Semi-nested and nested qPCR	Specific	3.0 × 10^2^ copies/mL		May be contaminated by PCR products
**HBV RNA**	RUO HBV RNA assay (Abbot)	Highly specific	44 IU/mL	Highly sensitive and specific; automated; high throughput	
	RACE-based methods	Specific	54 IU/mL	No additional steps in isolation procedure; sensitive; specific	
	Detection after DNase I treatment	Specific	66.7 IU/mL		Requires complicated isolation procedure (DNase I treatment and purification); time-consuming; allows enrichment of RNA compared to isolated DNA (does not eliminate all DNA)
